# Everyday Pedelec Use and Its Effect on Meeting Physical Activity Guidelines

**DOI:** 10.3390/ijerph17134807

**Published:** 2020-07-03

**Authors:** Hedwig T. Stenner, Johanna Boyen, Markus Hein, Gudrun Protte, Momme Kück, Armin Finkel, Alexander A. Hanke, Uwe Tegtbur

**Affiliations:** Institute of Sports Medicine, Hannover Medical School, 30625 Hanover, Germany; boyen.johanna@mh-hannover.de (J.B.); 1.markushein@gmail.com (M.H.); gudrun.protte@gawnet.ch (G.P.); kueck.momme@mh-hannover.de (M.K.); armin.finkel@gmx.de (A.F.); Hanke.Alexander@mh-hannover.de (A.A.H.); tegtbur.uwe@mh-hannover.de (U.T.)

**Keywords:** active transportation, heart rate, pedelec, e-bike, cycling

## Abstract

Pedelecs (e-bikes with electrical support up to 25 km·h^−1^) are important in active transportation. Yet, little is known about physiological responses during their everyday use. We compared daily pedelec (P) and bicycle (B) use to determine if pedelecs are a suitable tool to enhance physical activity. In 101 employees, cycling duration and intensity, heart rate (HR) during P and B were recorded via a smartphone app. Each recording period was a randomized crossover design and lasted two weeks. The ride quantity was higher in P compared to B (5.3 ± 4.3 vs. 3.2 ± 4.0 rides·wk^−1^; *p* < 0.001) resulting in a higher total cycling time per week for P (174 ± 146 min·wk^−1^) compared to B (99 ± 109 min·wk^−1^; *p* < 0.001). The mean HR during P was lower than B (109 ± 14 vs. 118 ± 17 bpm; *p* < 0.001). The perceived exertion was lower in P (11.7 ± 1.8 vs. 12.8 ± 2.1 in B; *p* < 0.001). The weekly energy expenditure was higher during P than B (717 ± 652 vs. 486 ± 557 metabolic equivalents of the task [MET]·min·wk^−1^; *p* < 0.01). Due to a sufficient HR increase in P, pedelecs offer a more active form of transportation to enhance physical activity.

## 1. Introduction

Insufficient physical activity and sedentary behavior are major risk factors for death from cardiovascular diseases, cancer, and diabetes worldwide [1]. The positive effects of physical activity have prompted leading health organizations to create activity guidelines to benefit health. However, globally, 25% of adults do not meet recommendations for physical activity [1]. Physical activity is important for all ages and should be integrated into daily life; for example, the workplace is a key setting where sedentary behavior can be reduced [2]. As active forms of transportation and commuting become more popular, a trip to and from the workplace offers the potential for increased productivity and a reduction in injuries and absenteeism [3,4]. The American College of Sports Medicine (ACSM) guidelines include cardiorespiratory exercise training of ≥150 min·wk^−1^ moderate-intensity (64–76% of maximum heart rate (HR_max_), or 3–5.9 MET) or ≥75 min·wk^−1^ vigorous-intensity (77–95% HR_max_ or 6–8.7 MET), or a combination of both, respectively [5].

In the course last decade, there has been a great deal of technological progress in the bicycle market with an increased number of pedelecs [6]. The number of pedelecs sold in Germany has steadily increased in recent years. In 2017, there were 720,000; in 2018, that number reached 980,000, which is an increase of 36% compared to the previous year [7]. Pedelecs are bicycles with electrical motor supports that can be gradually added. The motor is only active as long as the rider pedals, supporting a speed up to 25 km/h^−1^. At higher speeds, the work has to be done by the rider alone. The assumed advantages of a pedelec vs. a normal bicycle are shorter travel times, longer travel distances, and a higher number of trips. For longer commuting distances, challenging weather conditions, or the transportation of heavy loads, pedelecs are becoming increasingly popular [8,9].

However, current knowledge about the positive health effects of pedelecs usage remains sparse. Questionnaire studies show that pedelec trip distances were significantly longer compared with bicycles, but that physical activity levels were similar. This suggests that pedelec users may compensate for the lower exertion per kilometer by traveling for longer distances [10,11].

The physiological response of pedelec use in experimental studies shows increasing physical fitness, meeting physical activity recommendations determined by questionnaires, and in a laboratory setting for pedelec use [12]. Our goal is, however, to measure the physiological adjustments in everyday use.

We hypothesize that pedelec usage produces similar health-promoting effects compared with adult employees’ bicycle usage. Therefore, pedelecs are a suitable tool to enhance physical activity. Thus, the primary outcome was the total pedelec ride time minutes per week and the achieved heart rate, compared to bicycle rides.

Furthermore, we aimed to compare the results to ACSM guidelines for physical activities [5].

## 2. Materials and Methods

### 2.1. Study Design

The study was planned as an observational crossover study. To address the two different workplace settings of blue and white-collar workers, four different companies from Hanover, Germany, were chosen for the study. The employees were informed about the study via intranet, mail, and posters. Informative registration events were organized in the companies for interested employees. One hundred and nineteen volunteers were recruited. 101 (47 females, 54 males, age 43 ± 11 years, weight 82 ± 17 kg, for further details, see Table 1) were included in the study and met the following inclusion criteria: male and female workers between the ages of 18 and 65. Eighteen subjects were disqualified based on our exclusion criteria: diabetes, tumor diseases, coronary heart disease or arterial occlusive disease, unadjusted hypertension, an operation in the last eight weeks, a joint replacement six months prior to the study or suffering from other severe conditions counter indicating physical exercise. All participants were informed of the risks involved in this study and gave written informed consent prior to participation. The study protocol was approved by the ethics committee of Hannover Medical School (No. 6901-2015). The study was conducted in accordance with the Declaration of Helsinki.

### 2.2. Preliminary Physiological Testing

Before starting the study, all subjects visited the laboratory to undergo a brief physical examination by a physician. Additionally, body weight and body fat were determined using a direct-segmental multi-frequency bio-impedance scale (Inbody720, Biospace, Seoul, Korea). Height was measured using a stadiometer, and body weight was determined using a calibrated scale (Seca 764, seca GmbH & Co. KG, Hamburg, Germany). Body Mass Index (BMI) was calculated as the ratio of weight (in kg)/squared standing height (m^2^). Participants performed a graded exercise test on a cycle ergometer (Ergoselect 200, ergoline, Bitz, Germany). The test started at a workload of 20 W (for women) or 50 W (for men) for one minute and increased by 16.6 W every minute. Participants cycled until volitional exhaustion. Throughout the test, heart rate (HR) was recorded using a 12-channel-electrocardiogram (CardioSoft, GE Healthcare, Boston, USA) and respiratory gas exchange was measured breath-by-breath using an indirect calorimetry system (Masterscreen CPX, Becton Dickinson, Franklin Lakes, USA) HR and respiratory exchange values were averaged every 30 s. Peak oxygen consumption and maximum heart rate (HR_max_) was determined as the highest 30 s average during the exercise test.

### 2.3. Flow Protocol and Randomization

**Figure 1:**
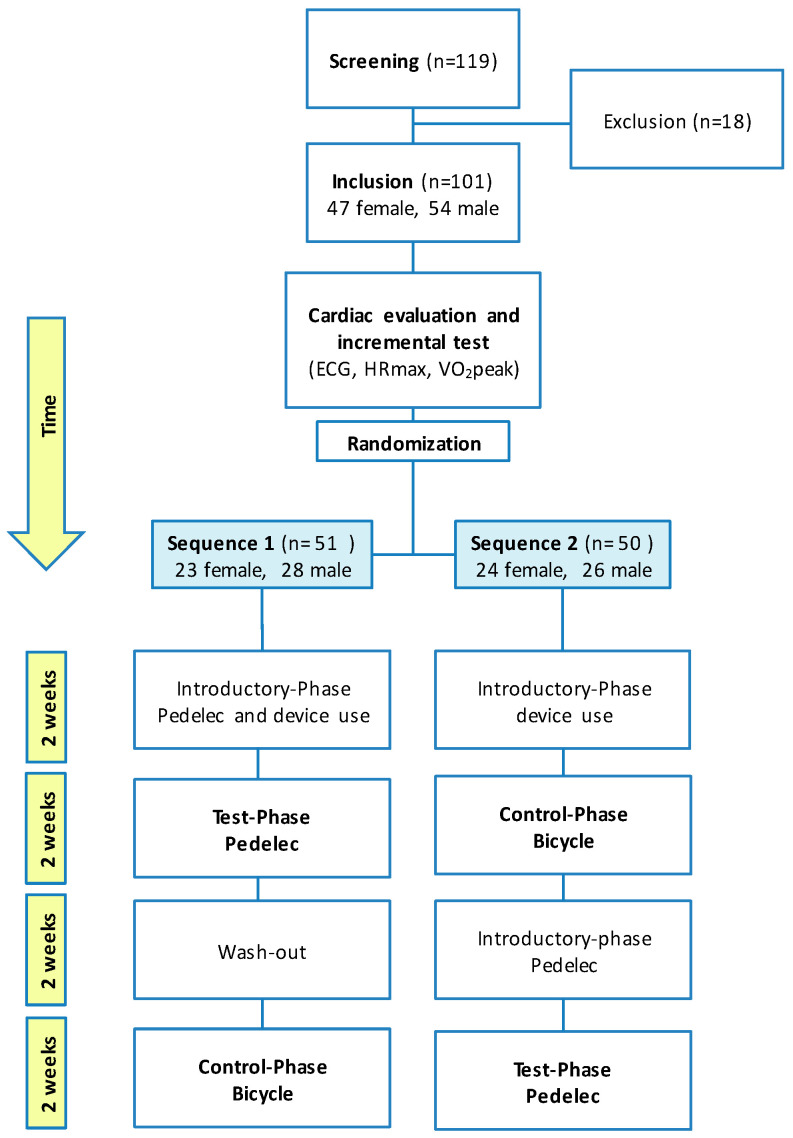
Flow chart of the study. Note the crossover design of sequences enabling each participant to be included in both groups, pedelec test group (P) and bicycle control group (B).

The observational flow protocol is displayed in Figure 1. All subjects were randomly assigned to two-week pedelec or bicycle use in a randomized crossover design. Participants were randomized in every company 1:1 into the two groups (sequence 1 or sequence 2) using a previously computer-based list of random numbers generated by a collaborator. Detailed information on the sequences pictured in Figure 1. The participants were informed about the group assignment, due to the nature of the used hardware (especially the pedelec motor) blinding was impossible.

According to the participants’ preferences, subjects were equipped with different pedelec models (city, trekking, mountain, carrier bikes). To eliminate the effect of curiosity, a two-week familiarization period with pedelecs was conducted before the recording period with the pedelec. To ensure that pedelec use did not have any effects on bicycle use, all subjects who started with the pedelec had a two weeks washout phase without pedelec before activities with the bicycle were recorded (Figure 1).

### 2.4. Trip Documentation via Monitoring Tools

Subjects recorded HR, duration, and perceived exertion of cycling digitally and in paper form during both observation periods. HR and Borg-scale were recorded via a smartphone app and a chest strap (Polar H7 Bluetooth smart, Polar, Kempele, Finland). The app was exclusively developed for this study (Inside m2m GmbH, Garbsen, Germany). Furthermore, an activity monitor (ActiGraph GT9X Link, Actigraph, Pensacola, FL, USA) was worn for assessing daily activity. The data were recorded via the app and transferred directly to an MHH database and saved on an internal university server. To detect changes in daily physical activity, participants were instructed to wear an activity monitor for the whole day during both periods. Activity monitor data were recorded in 1-min epochs. A wear time validation algorithm was used [13] and physical activity intensity was divided into two categories by a cut-off value of 1951 counts: sedentary to light (≤1951 counts) and moderate to very vigorous (≥1952 counts) [14]. The activity monitor had to be worn for ≥10 h day^−1^ to be considered a valid wear day [15]. Only subjects with ≥7 valid wear days in both observation periods were included in activity monitor analysis.

### 2.5. Measured Parameters

The duration of physical activity was recorded in one-minute intervals. Rating of perceived exertion (RPE) was given on the basis of the Borg-scale [16]. HR for a trip was calculated as average (without zeroes) over the duration of the trip. If more than 50% of the HR data were missing for a single trip, the trip was not taken into account for HR analysis. In 88 pedelec users (87%) and 62 bicycle users (61%), data sets were fully received.

### 2.6. Intensity Calculation

Regularly, MET calculation is based on oxygen consumption (VO_2_) during individuals’ exercise testing. During daily biking, we were not able to directly measure VO_2_. Thus, we calculated MET based on HR and VO_2_ derived from data of the graded exercise test. To estimate V0_2_ (mL/min/kg) for a given heart rate, the following equation was calculated using linear regression:

V02 = 0.23 × HR − 1.72(sex) + 0.05(age) − 8.63(1)

with the factor 1 for men (sex = 1) and factor 2 for women (sex = 2). Therefrom exercise intensity in MET was calculated by dividing VO_2_ with 3.5 mL/min/kg (equals 1 MET).

### 2.7. Statistical Analysis

Following a Kolmogorov–Smirnov test to verify normal distribution of the data, a student’s paired *t*-test was performed to analyze differences between P and B. The differences in intensity for all rides classified by %HR_max_ were analyzed with an unpaired student’s *t*-test. Values are presented as mean ± standard deviation (SD). An alpha of *p* < 0.05 was considered to be statistically significant. All analyses were carried out with the SPSS software package for Windows^®^ (Version 24, IBM Corp., Armonk, NY, USA).

## 3. Results

### 3.1. Trip Documentation/ Monitoring

**Figure 2:**
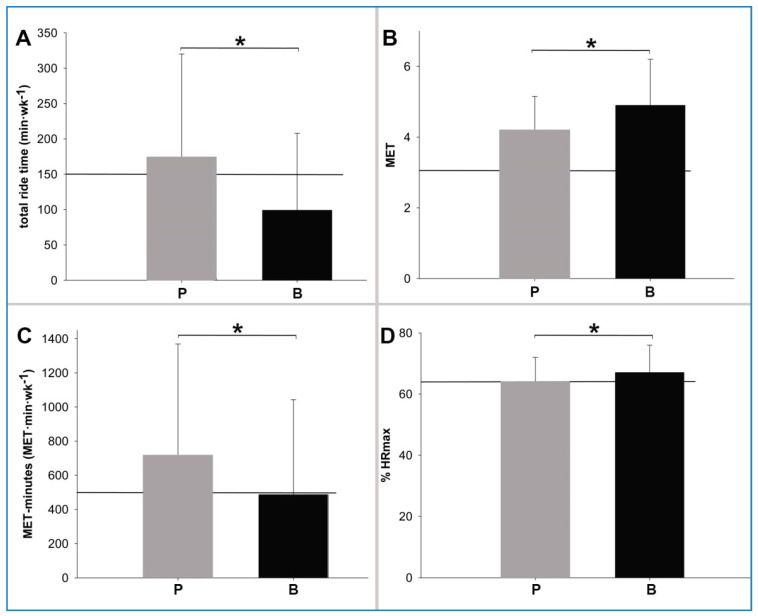
(**A**) Total ride time; (**B**) single trip intensity; (**C**) weekly metabolic rate; (**D**) mean intensity classified by %HR_max_; P—light grey: During pedelec use; B—dark grey: Bicycle use; The solid line shows the minimum requirements for moderate-intensity exercise as recommended by the ACSM; Significant differences between activity types (*p* < 0.05) are marked with a *.

Randomized sequencing did not affect ride time. Pedelec ride time, as well as bicycle ride time, did not show significant differences Pedelec time Sequence (S1 vs. S2: *p* = 0.26, bicycle time S1 vs. S2: *p* = 0.31). Total ride time was 35 ± 61% lower in B than in P (*p* > 0.001; Figure 2, Table 2). The number of trips was higher with P than B (5.3 ± 4.3 to 3.2 ± 4.0 trips·wk^−1^) (*p* < 0.001). Average trip duration did not differ between P and B (37.5 ± 23.5 to 40.3 ± 27.8 min/trip) (*p* = 0.45). During the two-week observation periods, 91% of the subjects used the provided pedelec and 69% used their own bicycle.

The distance from the participant’s home to their work was divided as follows: 1–3 km 7%, >3–5 km 13%, >5–10 km 15%, >10 km 18%, >20 km 31%, >50 km 16%.

Daily activity monitor wear time did not differ between the two observation periods (P 856 ± 72 min, B 864 ± 62 min, *p* = 0.28).

### 3.2. Measured Parameters

Daily activity time in the category sedentary to light did not show significant differences between the observation periods (P 805 ± 79 min, B 810 ± 77 min, *p* = 0.63) nor did moderate to vigorous activity (P 50.9 ± 35.0 min, B 54.3 ± 47.7 min, *p* = 0.50).

Mean HR was 8 ± 13% lower during P than B (109 ± 14 vs., *p* < 0.001) 118 ± 17 bpm. In both groups, %HR_max_ was in the range of ACSM recommendations [9] for both (Figure 2, Table 2).

Subjects perceived exertion was lower during P than during B (Borg-scale: 11.7 ± 1.8 to 12.8 ± 2.1, *n* = 70) (*p* < 0.001).

### 3.3. Calculated Intensity

Metabolic rate calculated from absolute HR during rides was lower in P than B, but sufficient to meet ACSM recommendations [9] for moderate-intensity in both (Figure 2). Energy expenditure expressed as the weekly metabolic rate was higher during P than during B (*p* < 0.01), meeting ACSM recommendations [9] in P but not in B (Figure 2, Table 2).

## 4. Discussion

Pedelec use requires similar physical effort as bicycle use, and therefore it is a suitable tool to enhance health-promoting physical activity. B achieved 35% less total time than P with 99 ± 109 min·wk^−1^. When riding P, the HF was only 8% lower than during B at 109 ± 14 bpm.

While both groups met ACSM criteria for moderate activity with respect to mean HR, total ride time was significantly less in B without achieving the recommended activity time of 150 min·wk^−1^.

However, taking MET and MET- min·wk^−1^ into account, our data clearly support that even though P usage is less exhausting than B (as described by lower MET) in our population, the total MET minutes per week were even higher in P as compared to B.

The results of our study suggest that pedelecs are used more often than normal bicycles in everyday use when no usage specifications are made. Similar observations have been made by Fyhri and Fearnley, who showed an increase of trips with the pedelec compared to the bicycle [17]. However, while more trips were executed, the average duration per trip showed no significant difference. We, therefore, assume that the participants mainly used the pedelecs for the same routes as the bicycles, but more often. This finding indicates that commuting and day-to-day tasks (e.g., grocery shopping), rather than additional recreational trips, were the main usage purpose.

Volume and intensity are the two parameters to evaluate if physical activity is sufficient to expect health promotion effects. Several other studies already showed that the heart rate during single trips with the pedelec can be classified as moderate by ACSM standards [17,18], and the pedelec was therefore assumed to be a promising mode meeting physical activity guidelines [18]. Our study is one of the first where no minimum riding requirements were made, and cardiorespiratory intensity during real-world pedelec usage patterns was analyzed in a large cohort.

In accordance with previous studies, our data confirm that the average heart rate during everyday pedelec use was significantly lower than during cycling, but was still high enough to be classified as moderate by ACSM standards [5]. This was supported by the calculation of the metabolic costs of P which also classify it as moderate intensity, but significantly less intense than B. The lower intensity was also perceived by the subjects, resulting in the reporting of lower exertion during P. This has been observed in other studies as well [18,19,20]. Lower perceived exertion has been reported to be a motivation to replace other modes of transportation (e.g., car or public transport) by the pedelec [21] and might, therefore, be the main reasons for the higher usage pattern we observed with the pedelec [17].

Importantly the weekly metabolic rate was significantly higher during P than B, showing higher energy expenditure while pedelecs were used. This shows that the volume compensates for the lower intensity observed during P. We can, therefore, assume that pedelecs may have beneficial health effects as reported previously [4,22] and we can confirm previous assumptions that the pedelec is an alternative to the bicycle for fulfilling health care guidelines [19].

The absence of differences in general activity between the two observation periods can be explained by the fact that cycling activity is not accurately recorded by the Actigraph activity monitor [23,24]. Therefore, neither pedelec use nor cycling affects the recorded total daily activity. The participants still wore the device all day to make sure every activity was recorded. The absence of changes in general activity indicates that using the pedelec did not influence other physical activities as observed in other activity promoting interventions [25]. Our findings are in accordance with other studies, which showed that the additional activity with the pedelec did not reduce other physical activities [22]. Our participants were very active in addition to the pedelec/bicycle use as activity counts for moderate to very vigorous activity were higher than the activities observed in other studies [22]. Hence, even in generally active people, the pedelec can be a health-promoting active transportation mode.

Pedelec use can help to meet the recommendations for physical activity, despite motor support. This shows that the integration of pedelecs in the form of active transportation to work is an important resource and should be supported by employers.

### Limitations

Although our study design included an introduction period where the participants got used to the pedelec, two weeks might have been not enough time to eliminate the effect of curiosity. Equipping the participants with GPS devices would have given our study further benefits in making pedelec and bicycle trips more comparable regarding speed, distance, and altitude. Recording the support level during the pedelec rides would have given further inside into the actual usage behavior as well. A further limitation is the relatively high number of participants who did not record heart rate during the trips due to technical limitations and based on measurements throughout the year with corresponding seasonal weather differences. For further studies, we, therefore, recommend easier to handle and more accepted options of heart rate recording, e.g., photoplethysmographic measurement on the wrist. As we only examined a two-week period, further research is necessary to clarify if the observed effects endure over longer time periods and a larger study population.

The examined population showed BMI and fat values slightly above desirable normal range but within average ranges in Germany, and thus, effects should be comparable to the general population.

## 5. Conclusions

On the basis of a real-world setting, our study showed that pedelecs could be a suitable method to enhance health-promoting physical activity in healthy adults. While the average physiological response is high enough to achieve beneficial adaptions, individual preconditions need to be taken into account for every pedelec user. As we only examined a two-week period, further research is necessary to clarify if the observed effects endure over longer time periods.

## Figures and Tables

**Table 1:** General subject information (*n* = 101). S1 and S2 display sequence association of S1: bicycle first vs. S2: pedelec first.

	All Mean ± SD	S1 Bicycle First Mean ± SD	S2 Pedelec First Mean ± SD	S1 vs. S2 *p*-Value
Gender (men/female)	54/47	27/24	27/23	0.915
Age (years)	43 ± 11	44 ± 12	42 ± 11	0.369
Height (cm)	174 ± 9	173 ± 9	175 ± 10	0.370
Bodyweight (kg)	82 ± 17	84 ± 19	81 ± 15	0.469
Body mass index (kg·m^−2^)	27.0 ± 4.8	27.6 ± 5.0	26.5 ± 4.4	0.218
Fat mass (%)	27 ± 9	28 ± 9	26 ± 9	0.381
Maximum Power output (W·kg^−1^)	2.6 ± 0.6	2.6 ± 0.7	2.6 ± 0.6	0.618
VO_2_ peak (mL·kg^−1^·min^−1^)	32 ± 8	32 ± 8	33 ± 8	0.674

VO_2_ peak = Peak oxygen consumption.

**Table 2:** Further results.

	P Mean ± SD	B Mean ± SD	*p*-Value
Number of trips (wk^−1^)	5.3 ± 4.3	3.2 ± 4.0	*p* < 0.001
Trip duration (min)	37.5 ± 23.5	40.3 ± 27.8	*p* = 0.45
Total ride time (min·wk^−1^)	174 ± 146	99 ± 109	*p* < 0.001

## References

[1] World Health Organization (WHO) Physical Activity. http://www.who.int/mediacentre/factsheets/fs385/en/.

[2] WHO (2018). Global Action Plan on Physical Activity 2018–2030: More Active People for a Healthier World.

[3] van Dongen J.M., Proper K.I., van Wier M.F., van der Beek A.J., Bongers P.M., van Mechelen W., van Tulder M.W. (2011). Systematic review on the financial return of worksite health promotion programmes aimed at improving nutrition and/or increasing physical activity. Obes. Rev. Off. J. Int. Assoc. Study Obes..

[4] de Geus B., De Smet S., Nijs J., Meeusen R. (2007). Determining the intensity and energy expenditure during commuter cycling. Br. J. Sports Med..

[5] Garber C.E., Blissmer B., Deschenes M.R., Franklin B.A., Lamonte M.J., Lee I.-M., Nieman D.C., Swain D.P. (2011). American College of Sports Medicine position stand. Quantity and quality of exercise for developing and maintaining cardiorespiratory, musculoskeletal, and neuromotor fitness in apparently healthy adults: Guidance for prescribing exercise. Med. Sci. Sports Exerc..

[6] Rose G. (2012). E-bikes and urban transportation: Emerging issues and unresolved questions. Transportation.

[7] Zweirad-Industrie-Verband, E.V Zahlen–Daten–Fakten zum Deutschen E-Bike-Markt 2018–2019. https://www.ziv-zweirad.de/fileadmin/redakteure/Downloads/PDFs/PM_2020_11.03._Fahrrad-_und_E-Bike_Markt_2019.pdf.

[8] MacArthur J., Dill J., Person M. (2014). Electric Bikes in North America: Results of an Online Survey. Transp. Res. Rec. J. Transp. Res. Board.

[9] Haustein S., Møller M. (2016). Age and attitude: Changes in cycling patterns of different e-bike user segments. Int. J. Sustain. Transp..

[10] Castro A., Gaupp-Berghausen M., Dons E., Standaert A., Laeremans M., Clark A., Anaya-Boig E., Cole-Hunter T., Avila-Palencia I., Rojas-Rueda D. (2019). Physical activity of electric bicycle users compared to conventional bicycle users and non-cyclists: Insights based on health and transport data from an online survey in seven European cities. Transp. Res. Interdiscip. Perspect..

[11] Nordengen S., Ruther D.C., Riiser A., Andersen L.B., Solbraa A. (2019). Correlates of Commuter Cycling in Three Norwegian Counties. Int. J. Environ. Res. Public. Health.

[12] Bourne J.E., Sauchelli S., Perry R., Page A., Leary S., England C., Cooper A.R. (2018). Health benefits of electrically-assisted cycling: A systematic review. Int. J. Behav. Nutr. Phys. Act..

[13] Choi L., Liu Z., Matthews C.E., Buchowski M.S. (2011). Validation of accelerometer wear and nonwear time classification algorithm. Med. Sci. Sports Exerc..

[14] Freedson P.S., Melanson E., Sirard J. (1998). Calibration of the Computer Science and Applications, Inc. accelerometer. Med. Sci. Sports Exerc..

[15] Troiano R.P., McClain J.J., Brychta R.J., Chen K.Y. (2014). Evolution of accelerometer methods for physical activity research. Br. J. Sports Med..

[16] Borg G. (1990). Psychophysical scaling with applications in physical work and the perception of exertion. Scand. J. Work. Environ. Health.

[17] Fyhri A., Fearnley N. (2015). Effects of e-bikes on bicycle use and mode share. Transp. Res. Part Transp. Environ..

[18] Gojanovic B., Welker J., Iglesias K., Daucourt C., Gremion G. (2011). Electric bicycles as a new active transportation modality to promote health. Med. Sci. Sports Exerc..

[19] Simons M., Van Es E., Hendriksen I. (2009). Electrically assisted cycling: A new mode for meeting physical activity guidelines?. Med. Sci. Sports Exerc..

[20] Sperlich B., Zinner C., Hébert-Losier K., Born D.-P., Holmberg H.-C. (2012). Biomechanical, cardiorespiratory, metabolic and perceived responses to electrically assisted cycling. Eur. J. Appl. Physiol..

[21] Paul F., Bogenberger K. (2014). Evaluation-method for a Station Based Urban-pedelec Sharing System. Transp. Res. Procedia.

[22] Peterman J.E., Morris K.L., Kram R., Byrnes W.C. (2016). Pedelecs as a physically active transportation mode. Eur. J. Appl. Physiol..

[23] Herman Hansen B., Børtnes I., Hildebrand M., Holme I., Kolle E., Anderssen S.A. (2014). Validity of the ActiGraph GT1M during walking and cycling. J. Sports Sci..

[24] Steeves J.A., Bowles H.R., McClain J.J., Dodd K.W., Brychta R.J., Wang J., Chen K.Y. (2015). Ability of thigh-worn ActiGraph and activPAL monitors to classify posture and motion. Med. Sci. Sports Exerc..

[25] Mansoubi M., Pearson N., Biddle S.J.H., Clemes S.A. (2016). Using Sit-to-Stand Workstations in Offices: Is There a Compensation Effect?. Med. Sci. Sports Exerc..

